# Spatial analysis of drug resistant tuberculosis (DRTB) incidence and relationships with determinants in Rio de Janeiro state, 2010 to 2022

**DOI:** 10.1371/journal.pone.0321553

**Published:** 2025-05-02

**Authors:** Paula Cristina Pungartnik, Paulo Victor de Souza Viana, Jefferson Pereira Caldas dos Santos, Laylla Ribeiro Macedo, Thais Zamboni Berra, Natália Santana Paiva

**Affiliations:** 1 Institute of Collective Health Studies, Federal University of Rio de Janeiro (UFRJ), Rio de Janeiro, Brazil; 2 Professor Hélio Fraga Reference Center, Sergio Arouca National School of Public Health, Oswaldo Cruz Foundation (FIOCRUZ), Rio de Janeiro, Brazil; 3 Center for Innovation in Biodiversity and Health, Oswaldo Cruz Foundation (FIOCRUZ), Rio de Janeiro, Brazil; 4 Sergio Arouca National School of Public Health, Oswaldo Cruz Foundation (FIOCRUZ), Rio de Janeiro, Brazil; 5 Ribeirao Preto School of Nursing, University of Sao Paulo (EERP/USP), São Paulo, Brazil; UFSJ: Universidade Federal de Sao Joao del-Rei, BRAZIL

## Abstract

**Background:**

The aim of this study was to assess the spatial distribution of drug-resistant tuberculosis (DRTB) cases in Rio de Janeiro state and its association with demographics, socioeconomic and health determinants.

**Methods:**

An ecological study based on real-world DRTB data from 2010 to 2022, in the Rio de Janeiro state, using data from the Special Tuberculosis Treatment Information System (SITE-TB) and demographic census. Crude incidence rates (CIR) of DRTB per 100,000 inhabitants and smoothed rates through the Global and Local Empirical Bayesian (BEG and BEL) methods were calculated. Spatial autocorrelation was explored using Moran’s I statistic, Local Indicators of Spatial Association (LISA), and the Getis-Ord statistics. The SCAN method was also used to identify spatial-time clusters. To analyze the association of DRTB and determinants, we used LISA bivariate for spatial correlation and four explanatory statistical models were listed.

**Results:**

From 2010 to 2022, 2,709 new cases of DRTB were reported (CIR 16.9/100,000 inhabitants). The municipalities in the metropolitan region of Rio de Janeiro state had the highest rates. Despite 41% of municipalities reporting no new cases, BEG and BEL suggested higher rates than CIR, indicating underreporting. Spatial heterogeneity was observed, and spatial and spatial-temporal clusters and hotspots were detected in metropolitan region. Family health strategy coverage was identified as protection factor, however a not expected negative spatial autocorrelation between CIR and health strategy coverage, primary care and healthcare agent coverage was found. The variables identified as risk factors were population aged ≥18 years old with Elementary School completed (OR:1.10; CI95%:1.04–1.16), demographic density (OR: 1.00; CI95%:1.00–1.01), HIV-TB coinfection (OR: 1.18; CI95%:1.06–1.31).

**Conclusion:**

The identification of areas of risk for DRTB, spatial correlation and association between incidence and determinants, demonstrates that the DRTB transmission dynamics is related to the perpetuation of social inequality and urban spatial organization.

## Introduction

Tuberculosis (TB) is an infectious and preventable disease that affects more than 10 million people worldwide annually. Despite being a preventable and curable disease, TB has once again become the leading cause of death from a single infectious agent, surpassing even COVID-19 in recent years [1,2]. Brazil is on the list of 30 high-burden countries, with significant burdens of TB and TB-HIV co-infection [1]. The incidence of TB in the country has been a growing concern since 2016, with 80,012 new cases reported in 2023, which corresponds to an incidence rate of 37.3 cases per 100,000 people [3]

This rise in TB cases has also led to an increase concern of drug-resistant tuberculosis (DRTB), which is directly related to the proper management of drug-sensitive TB. Additionally, other factors have been described as DRTB determinants, such as socio-economic, spatial, demographic, cultural, clinical and environmental factors [4–7]. Health access and the quality of services, especially in terms of detecting new cases of the disease, are also a major factor described in studies [7–9].

The complex, long-term, and expensive treatment of DRTB is recognized as a significant public health issue, representing one of the greatest challenges in managing TB globally [10]. The emergence of drug resistance presents an additional risk of spreading resistant strains, leading to the primary infection of the disease [10]. Currently, studies in Brazil and other countries, such as China, are describing a higher incidence of primary DRTB than the development of drug resistance during TB management [11–13].

Although Brazilian efforts to TB and DRTB control [14], the incidence of DRTB is increasing. Between 2015 and 2023, 17,200 new cases of DRTB were reported in the country, with 1,060 diagnosed in 2023 [3]. In 2023, the state of Rio de Janeiro had the third highest TB incidence among Brazilian states and the highest proportion of DRTB cases in the country, at approximately 25% [15,16]. The state displays significant socio-spatial disparities and geographic complexity by high population density and disorderly urban growth [17,18]. The Metropolitan area of the state, encompassing major urban centers with substantial concentrations of subnormal living areas, accounts for approximately 77% of TB cases, reflecting its urban complexity [16]. Additionally, despite having the one of the best TB detection rates, Rio de Janeiro shows fluctuation in its healthcare system coverage over time, imposing further challenges for the effective control of TB and DRTB [14,19,20].

Spatial analysis based on the recognition that the spread of a phenomenon across space is profoundly influenced by human mobility patterns, has allowed a more comprehensive dynamics involved in disease transmission and the presence of highly localized mobility clusters [21]. Previous spatial analyses of DRTB have demonstrated heterogeneous spatial distribution and clusters in specific geographical areas associated with urban areas population density [5,6,22,23]. In Brazil, although the large scales of TB articles, studies related to DRTB are scarce. Some spatial analyses were conducted in other states [7,12,24,25], however so far, the spatial pattern of the disease in Rio de Janeiro is not known. The aim of this study was to assess the spatial distribution of DRTB cases in Rio de Janeiro state and its association with demographics, socioeconomics, and health determinants.

## Materials and methods

### Study design

**Fig 1:**
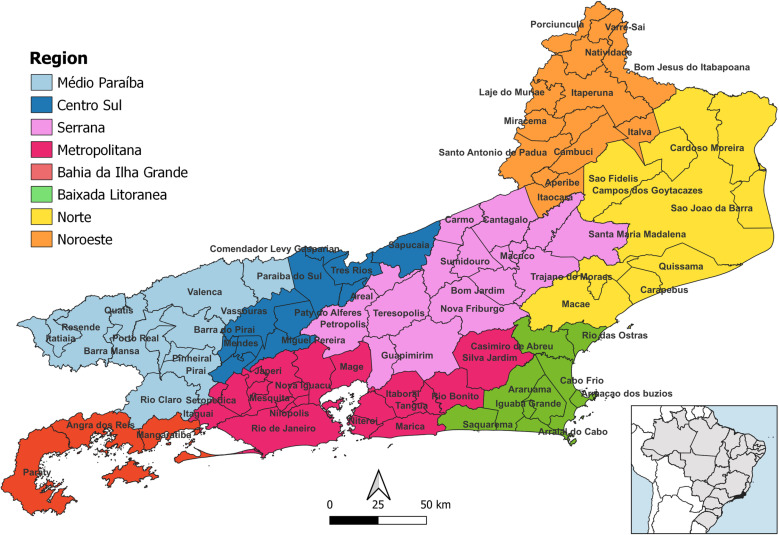
Rio de Janeiro state divided by region and municipalities. Shapefile publicly available source: *IBGE Malha Municipal* - territorial mesh.

An ecological study was conducted based on real-world data of new reported cases of DRTB between 2010 and 2022, in Rio de Janeiro state, and its relationship with demographics, socioeconomics and healthcare services indicators. Rio de Janeiro state is located in the southeast region of Brazil, occupies an area of approximately 43,750 km² and a population of 16,054,524 inhabitants, with a population density of 366.96 inhabitants/km^2^ [26]. It is divided into 8 health regions and 92 municipalities (Fig 1). These regions are divided according to the similarities in the characteristics of each municipality, based on the decentralization process of transferring resources and responsibilities for executing Health Surveillance actions at the municipal level [27].

### Population and data sources

The study population consisted of new cases of DRTB reported in the Tuberculosis Special Treatment Information System (SITE-TB) from 2010 to 2022. Non-nominal records of new DRTB cases reported in SITE-TB in Rio de Janeiro were requested through Fala.BR - Integrated Ombudsman and Information Access Platform (falabr.cgu.gov.br) from the Ministry of Health. Cases classified as ‘change of diagnosis’ for treatment outcome and/or cases where the municipality of residence was listed as ‘ignored’ or ‘missing’ were excluded from the study.

To analyze factors associated with the incidence of DRTB, indicators of demographics, socioeconomic and healthcare services access were calculated/selected, from open-source population data from the Demographic Census of municipalities by the Brazilian Institute of Geography and Statistics (IBGE) and Notifiable Diseases Information System (SINAN). During the study analysis, the 2022 Demographic Census was still being developed, and some information was not yet available. In cases where certain indicators were unavailable, data from the 2010 Demographic Census were used.

The socioeconomics variables selected were Municipal Human Development Index (MHDI), Gini index, Percentage of population aged ≥18 years old with Elementary School completed, Percentage of college degree completed population, Percentage of poor population and Proportion of people vulnerable to poverty. To evaluate demographics indicators, we selected Demographic density, Household crowding density, Urban areas density, Percentage of black/brown ethnicity and Percentage of male gender. The selected healthcare indicators were Percentage of TB treatment loss to follow up, Percentage of HIV-TB coinfection, Percentage of drug susceptibility testing, Family health strategy coverage, Primary care coverage and Healthcare agents’ coverage. Three different indicators related to healthcare coverage were included, as the Public Health System (*Sistema Único de Saúde* – SUS) has several services and metrics to measure its quality [28]. The calculation of each indicator is described in detail in S1 Table. The aggregated data of DRTB and the publicly available data of the indicators used in this analysis are available in S2 Table.

### Data analysis

To analyze the spatial distribution of DRTB incidence rates, the crude incidence rates (CIR) accumulated were calculated by municipality in Rio de Janeiro state for the period from 2010 to 2022. To minimize the instability caused by the random fluctuation in CIR, the rates were smoothed by the Empirical Bayes estimation [29]: The Global Empirical Bayesian (BEG) method, which estimates the weighted average between the locality’s CIR and the region’s global rate, and the Local Empirical Bayesian (BEL) method, which uses a neighborhood matrix by contiguity, adjusting the CIR with a “neighborhood” average (border municipalities) instead of a global average [14].

Spatial autocorrelation was evaluated by Moran’s I and the Getis-Ord statistic. The global Moran’s I test the presence, strength, and direction of spatial autocorrelation of the total area observed and is estimated from a pre-established neighborhood parameter. It can range from −1 to +1, the negative values being indicators of negative or inverse autocorrelation, and the positive, of spatial aggregation or direct correlation. The level of significance for spatial autocorrelation was p < 0.05 [30]. At a local scale, the local indicator of spatial association (LISA) for the detection of regions with significant spatial correlation and clustering measures and the Getis-Ord statistics to identify the locations of hot-spots were explored [31,32].

The Getis-Ord Gi* technique indicates local association, considering the values for each census sector of the municipality based on a neighborhood matrix [32]. This analysis also generates a z-score for statistically significant census tracts. The higher the z-score, the more intense the clustering of high values (Hotspot), while the lower the value, the more intense the clustering of low values or lower occurrence of the event (Coldspot). In addition to the z-score, the p-value and significance level (Gi-Bin) are provided. Gi-Bin values identify statistically significant hot and cold spots. Values range from ±3 and reflect statistical significance with a 99% confidence level, ±2 with a 95% confidence level, ±1 with a 90% confidence level, and zero is not statistically significant [32]. The “Incremental Spatial Autocorrelation” (ISA) tool in ArcGis 10.5 software was used to obtain the best distance at which spatial clusters are most pronounced [33].

Additionally, to verify whether the geographic clustering of DRTB was caused by random variation or not during a period of time, the space-time scan statistical analysis proposed by Kulldorff and Nagarwalla [34] was performed with Poisson probability distribution. The spatial-time scanning analysis is based the cylindrical window moved in space and time, with the base of the column corresponding to geographic region and the height of the column corresponding to time. For each scanning window, the theoretical incidence can be calculated according to the total incidence and the number of people in the window, and then the logarithmic likelihood ratio (LLR) is constructed by using the actual incidence and expected incidence inside and outside the scanning window respectively. The window with the highest LLR is defined as the most likely cluster area, and other clusters with statistically significant LLR were defined as the secondary potential clusters. Statistic imposes a window, which varies continuously in size from zero to some upper limit, in this study defined as 50% of the total population. In order to test the null hypothesis that no difference exists in the relative risk (RR) of DRTB between the neighborhoods, we used the Monte Carlo approach with 9,999 repetitions, with significance set at 0.05. Overlap was not permitted [35].

Regarding the association between the occurrence of DRTB and socioeconomics, demographics and healthcare indicators, first a spatial correlation between CIR and indicators was performed through a bivariate cluster analysis including bivariate Global Moran’s I and bivariate LISA (BiLISA), as the extension of univariate cluster analyses. BiLISA was carried out considering the bivariate Global Moran’s I statistically significant (p < 0.05) [31].

Second, the indicators were considered as explanatory variables to build an explanatory model based on scientific literature and related to the occurrence of DRTB. For the model, we considered the number of DRTB cases according to municipalities in Rio de Janeiro as the dependent variable, and the socioeconomics, demographics and healthcare indicators were established as independent variables. Before inserting the variables into the multiple model, multicollinearity was verified using the Variance Inflation Factor (VIF), and those with values greater than 10 were subtracted from the statistical model [36].

Considering the dependent variable (number of DRTB cases) as a count, four explanatory statistical models were listed with the following probability distributions: Poisson, Negative Binomial (NB), Zero-inflated Poisson (ZIP) and Zero-inflated Negative Binomial (ZINB). The objective of using four different models was to verify the best adequacy of the modeling given the nature of the data used [37].

The best model was weighted based on the highest values of the logarithm of the likelihood (Log likelihood) of the model and the lowest values of the Akaike Information Criterion (AIC). It is worth noting that for the model with the best comparison parameters, the Odds Ratio (OR) and respective 95% CI were calculated. Type I error was set at 5% as statistically significant (p < 0.05).

The shapefile of the Rio de Janeiro state map in 2023 was obtained from IBGE *Malha Municipal* (https://www.ibge.gov.br/geociencias/organizacao-do-territorio/malhas-territoriais/15774-malhas.html). For this study, an adjustment was made to the current cartographic network of the state of Rio de Janeiro, connecting the municipalities of Rio de Janeiro and Niterói. Although these municipalities do not share direct geographical boundaries, they are connected by a bridge, which facilitates traffic and interaction between them. This connection may impact spatial analyses that are based on neighborhood contiguity, such as BEL estimates, and Getis-Ord Gi*.

Data management and statistical models were estimated using using *Software R* version 4.3.0 (https://cran.r-project.org/). SCAN method was performed by *software* SaTScan package 9.6.1 (https://satscan.org/). The maps were developed in *software* QGIS version 3.18.1.

### Ethical statement

The study protocol was reviewed and approved by Research Ethics Committee of the Institute for Studies in Public Health at the Federal University of Rio de Janeiro (IESC/UFRJ), under number 6.081.205, with dispensation of the Informed Consent Form for the use of anonymized data with non-open access from SITE-TB.

## Results

**Fig 2:**
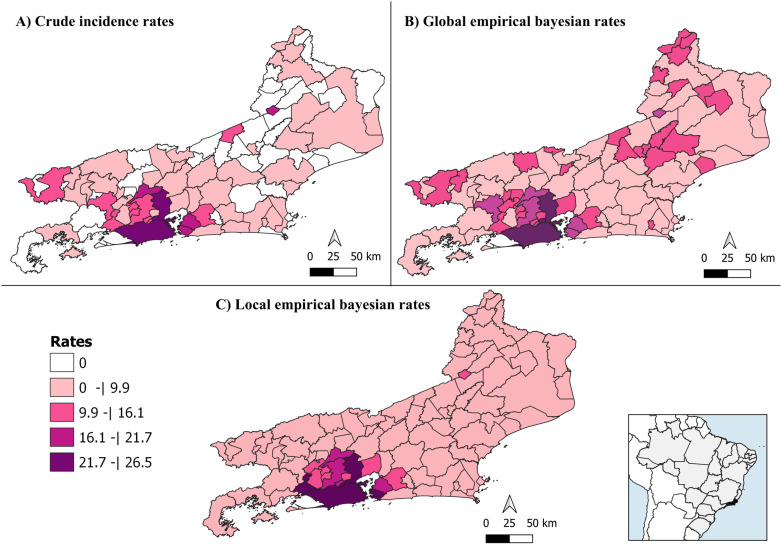
Spatial distribution of DRTB (A) crude incidence rates, and smoothed rates by (B) Global empirical Bayesian method and (C) Local empirical Bayesian method per 100,000 inhabitants per municipalities of Rio de Janeiro state, from 2010 to 2022. Shapefile publicly available source: IBGE Malha Municipal - territorial mesh.

A total of 2,709 new cases of DRTB were reported in Rio de Janeiro state from 2010 to 2022, with a CIR of 16.9 cases per 100.000 inhabitants. Higher CIR were concentrated in the Metropolitan region of Rio de Janeiro state, mainly in the municipalities of Rio de Janeiro (26.4/100.000 hab.) and São João de Meriti (25.2/100.000 hab.), followed by Duque de Caxias (24.5/100.000 hab.), Miguel Pereira (18.8/100.000 hab.) and Niterói (18.3/100.000 hab.). It is important to mention that 38 municipalities (41%) did not report new cases of DRTB spread in all regions of the state (Fig 2 and S3 Table).

Smooths rates estimated by BEG and BEL model, demonstrated average and median higher than CIR (Tables 1 and S3). These smoothing estimates indicated positive incidence rates even for municipalities without reported cases, reflecting the tendency to approximate the state’s average rate in the BEG model and the influence of the spatial pattern of neighborhood boundaries in the BEL model. Although 41% of the municipalities did not reported cases of DRTB, they all shared borders with municipalities that had a CIR greater than 1.4/100,000 inhabitants (Fig 2 and S3 Table). BEL estimates highlighted the concentration of DRTB in the metropolitan region. On the other hand, for BEG map, in addition to metropolitan region, highlighted the northern and Serrana regions as areas with high rates (Fig 2).

**Table 1:** Descriptive statistical incidence rates of DRTB new cases in Rio de Janeiro state, Brazil, from 2010–2022.

Estimative	Mean (SD)	Median (IQR)	Range (Min; Max)
CIR	5,09 (6.46)	2,50 (0.00; 7.80)	0.00; 26.47
BEG	9,84 (4.55)	8,92 (6.52; 12.28)	2.79; 26.44
BEL	6,65 (5.85)	5,19 (2.59; 7.47)	0.24; 26.41

**Fig 3:**
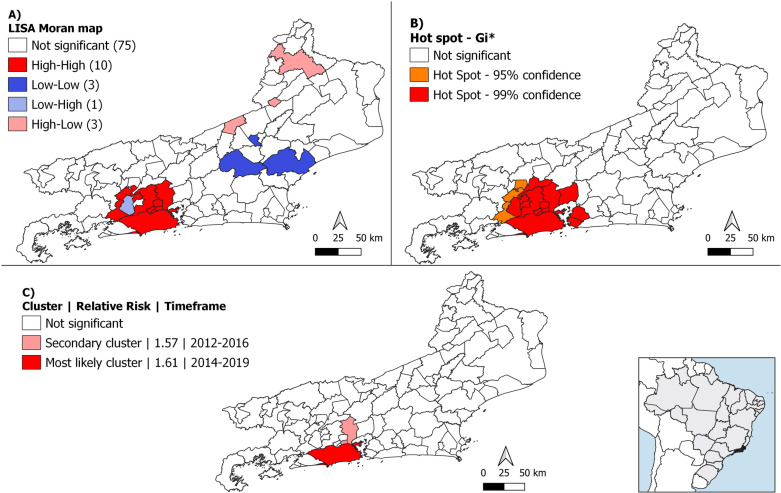
Spatial clustering of DRTB in Rio de Janeiro state per municipality, from 2010 to 2022, based on Local Spatial correlation – LISA (A) Getis-Ord Gi* statistic (B), and Spatio-time Cluster (C). Shapefile publicly available source: *IBGE Malha Municipal* - territorial mesh.

Moran I. demonstrated a statistically significant spatial correlation (0.44; p-value < 0.01) of DRTB in Rio de Janeiro state. Four disease aggregation areas by LISA analysis were identified. The municipalities in the metropolitan region in high-high aggregation area were Rio de Janeiro, Niterói, Belford Roxo, São João de Meriti, Mesquita, Nilópolis, Duque de Caxias, Nova Iguaçu, Paracambi, Japeri and São Gonçalo, indicating that high risk of disease cases was associated with high risk of disease in neighbors. In contrast, although the municipality of Seropédica is located in the metropolitan area, it was classified as low-high, reflecting a low CIR and higher CIR in the surrounding areas (Fig 3).

In the Getis-Ord Gi* technique, more municipalities were identified as hot spots at a 99% confidence interval (CI) compared to the high-high cluster from LISA. The same municipalities identified include Rio de Janeiro, Niterói, Belford Roxo, São João de Meriti, Mesquita, Nilópolis, Duque de Caxias, Nova Iguaçu, Japeri, São Gonçalo, as well as Mage, Miguel Pereira, Queimados, and Seropedica. Additionally, hot spots at a 95% CI were observed in the municipalities of Engenheiro Paulo de Frontin, Itaguaí, and Paracambi (Fig 3).

Space-time clustering analysis by SaTSCan identified two statistically significant clusters (p < 0.05) localized in the metropolitan region. The most likely cluster, with RR 1.61, appeared from 2014–2019 and was composed by Rio de Janeiro, and the secondary cluster, composed of Duque de Caxias and São João de Meriti, from 2012–2016 presented a RR of 1.57 (Table 2 and Fig 3).

**Table 2:** Spatial-time clusters of TBDR in the Rio de Janeiro state, from 2010 and 2022.

Cluster type	Municipality	Time frame	Cluster population	Observed Cases (n)	Expected cases	Crude incidence rate (per 100,000 inhabitants)	LLR	RR	P-value
Most likely	Rio de Janeiro	2014-2019	6,205,555	866	613.45	2.3	61.9	1.61	<0.001
Secondary cluster	Duque de Caxias,	2012-2016	1,247,934	158	102.87	2.5	13.3	1.57	<0.001
	São João de Meriti								

RR - Relative risk; LLR - Logarithmic Likelihood Ratio.

The Bivariate Global Moran I. between CIR and indicators revealed a positive spatial autocorrelation statistically significant for demographic density indicator (Moran I. = 0.46; p < 0.01). The indicators Urban areas density (Moran I. = 0.38), Household crowding density (Moran I. = 0.35), Percentage of population aged ≥18 years old with Elementary School completed (Moran I. = 0.26), Percentage of black/brown ethnicity (Moran I. = 0.21), HIV-TB coinfection (Moran I. = 0.18), MHDI (I. Moran = 0.17) also had a positive and statistically significant spatial correlation with DRTB CIR, but lesser. On the other hand, the indicators Primary care coverage (Moran I. = -0.26), Family health strategy coverage (Moran I. = -0.24), Percentage of male gender (Moran I. = -0.21) and Healthcare agents’ coverage (Moran I. = -0.2) presented a negative spatial correlation (Table 3).

**Table 3:** Bivariate global Moran I between crude incidence rate of DRTB and demographics, socioeconomics and healthcare services indicators in the Rio de Janeiro state, from 2010 to 2022.

Dimension	Indicator	Moran I	P-Valor
**Socioeconomics**	**MHDI**	0.17	**0.027**
	**GINI index**	-0.08	0.326
	**population aged ≥18 years old with Elementary School completed**	0.26	**0.010**
	**College degree completed population**	0.07	0.335
	**Proportion of poor population**	-0.11	0.157
	**Vulnerable to poverty**	-0.12	0.13
**Demographics**	**Household crowding density**	0.35	**< 0.001**
	**Proportion of black/brown ethnicity**	0.21	**0.006**
	**Proportion of male gender**	-0.21	**0.007**
	**Demographic density**	0.46	**< 0.001**
	**Urban areas density**	0.38	**0.001**
**Healthcare service**	**Proportion of TB treatment interruption**	0.13	0.094
	**Drug susceptibility testing**	-0.1	0.160
	**HIV-TB coinfection**	0.18	**0.022**
	**Primary care coverage**	-0.26	**< 0.001**
	**Family health strategy coverage**	-0.24	**0.002**
	**Healthcare agents’ coverage**	-0.2	**0.007**

**Fig 4:**
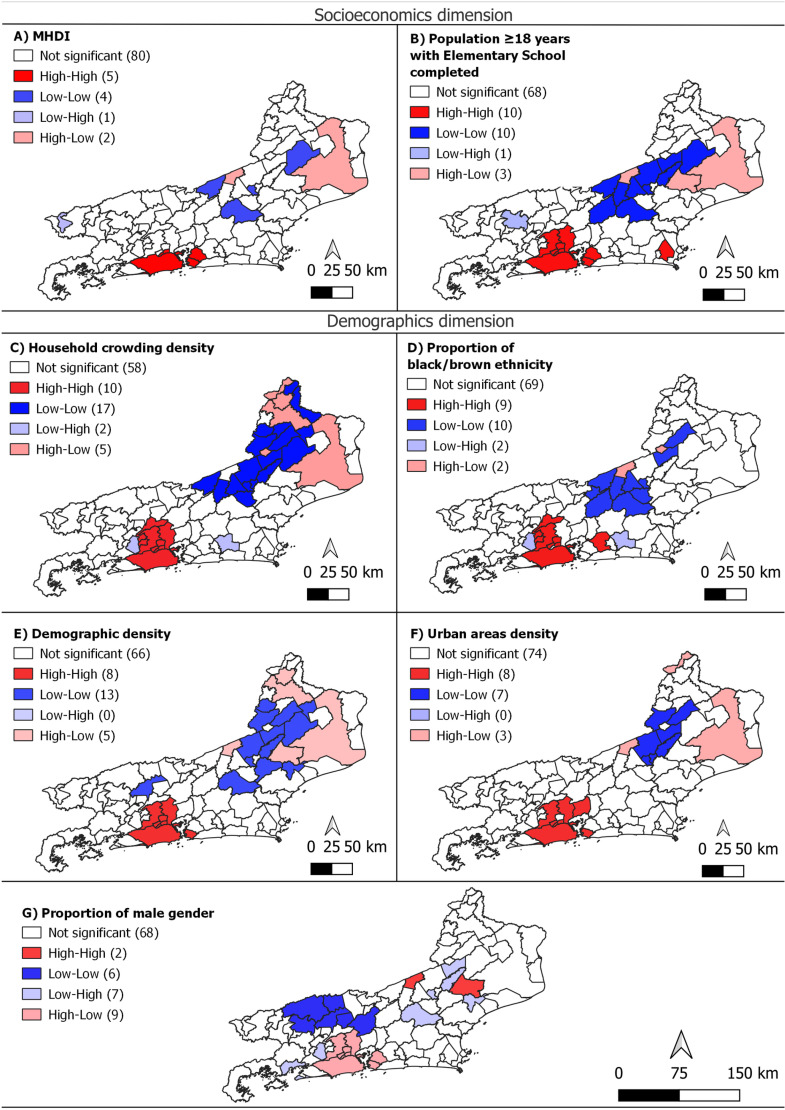
Bivariate analysis (LISA) of the crude incidence rate of DRTB and socioeconomics and demographics indicators with statistically significant spatial autocorrelation, municipalities of the state of Rio de Janeiro.

**Fig 5:**
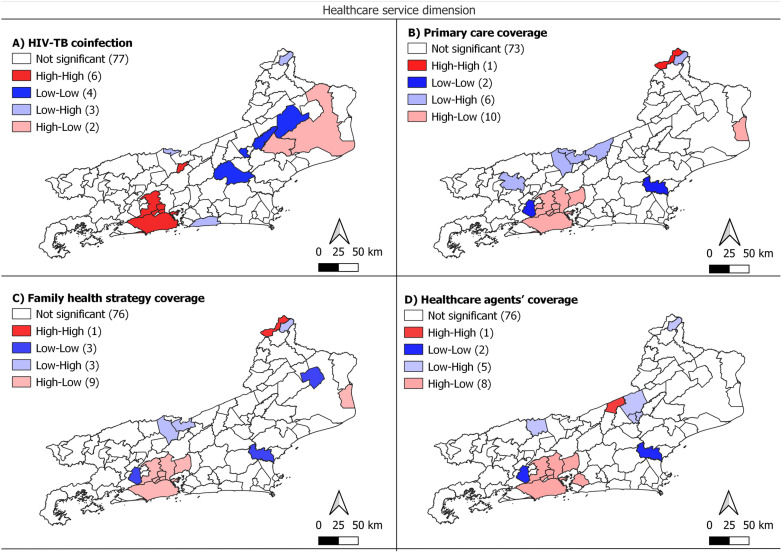
Bivariate analysis (LISA) of the crude incidence rate of DRTB and Healthcare services indicators with statistically significant spatial autocorrelation, municipalities of the state of Rio de Janeiro.

The BiLISA cluster map demonstrated the cluster patterns of spatial correlations between DRTB CIR and indicators. The high risk of DRTB had a positive spatial correlation with high CIR and high indicators (High-High cluster) Demographic density, Household crowding density, MHDI, Percentage of black/brown ethnicity, Percentage of population aged ≥18 years old with Elementary School completed, Urban areas density and HIV-TB coinfection, in the metropolitan region. Municipalities in the Serrana and Northwest regions of Rio de Janeiro state, presented Low-Low cluster, indicating low CIR of DRTB and low indicators values. However, an unexpected inverse relationship between variables in some municipalities was observed when High-Low and Low-High clusters appears, presenting high rates in areas with low indicators and low rates in areas with high indicators, respectively (Figs 4 and 5). The healthcare service indicators: Family health strategy coverage, Primary care coverage and Healthcare agents’ coverage, had Bivariate Moran I. negative and inverse than expected, with spatial correlation High-Low in the metropolitan region (Fig 5).

Table 4 presents the four models tested and their AIC and Log Likelihood values used to choose the best model. Of the models tested, the ZINB probabilistic distribution was the one that best suited the nature of both dependent variables, this was observed due to its highest Log Likelihood values (211.2) and lowest AIC values (438.4).

**Table 4:** Comparison of the metrics used to choose the best model.

	AIC	Log Likelihood
Poisson	1231.9	-601.9
Negative Binomial (NB)	438.4	-211.2
Zero-Inflated Poisson (ZIP)	1356.5	-656.2
Zero-Inflated Negative Binomial (ZINB)	443.6	-216.8

Associations were identified with the number of DRTB cases and the Percentage of population aged ≥18 years old with Elementary School completed (OR: 1.10; CI95%: 1.04–1.16), Demographic density (OR: 1.00; CI95%: 1.00–1.01), Percentage of HIV-TB coinfection (OR: 1.18; CI95%: 1.06–1.31) and an inverse association with Family health strategy coverage (OR: 0.97; CI95%: 0.95–0.98), according to Table 5.

**Table 5:** Negative Binomial (NB) explanatory model considering the number of cases of Drug-resistant Tuberculosis and sociodemographic variables.

Variable	Estimate	P-value	Odds Ratio (OR)	CI 95%
Intercept	-5.09	0.01*	0.00	0.01-0.29
Gini index	3.55	0.37	34.87	0.03-1.16
Percentage of population aged ≥18 years old with Elementary School completed	0.09	<0.01*	1.10	1.04-1.16
Demographic density	<0.01	<0.01*	1.00	1.00-1.01
Percentage of HIV-TB coinfection	0.17	<0.01*	1.18	1.06-1.31
Family health strategy coverage	-0.03	<0.01*	0.97	0.95-0.98

## Discussion

This retrospective study provided the first and detailed overview of the spatial high-risk areas of DRTB in the Rio de Janeiro state, demonstrating heterogeneity in the distribution of DRTB cases, with significant high-risk clusters over time and positive association with determinants.

Areas with higher DRTB CIR were shown to be concentrated in the metropolitan region, which accounts for 70% of TB cases in the state [16]. Clusters and hotspots were concentrated in Rio de Janeiro, Duque de Caxias, Niterói, São João de Meriti, São Gonçalo and Nova Iguaçu, the most populous municipalities in the state. This raises concerns due to the high population density and mobility, which may contribute to disease transmission. In addition to well-known spatial inequality, the state of Rio de Janeiro is notable for having the highest socioeconomic inequality among Brazilian states [20,27,38,39]. The municipalities identified as DRTB clusters are particularly characterized by a high concentration of slums [40].

The positive spatial autocorrelation between DRTB incidence rates and urban density areas reinforces the relationship between TB and DRTB and urban spatial organization. It highlights the necessity of prevention and control strategies in urban areas, where the risk of transmission is exacerbated [5,6,22,23,25], especially with the increase of primary DRTB infection, which accounts for approximately 60% of cases [11–13]. Another well-established indicator in the context of disorderly expansion of urban cities, is the household crowding density, which also demonstrated spatial correlation with DRTB incidence. This indicator is directly associated with the increased risk of spread diseases, especially in settings that facilitate the transmission through the air [38,41].

These results, combined with study demonstrating an upward trend in DRTB cases across all Brazilian regions since 2016, particularly in the state of Rio de Janeiro, emphasize the importance of considering human mobility patterns and the dynamics of spatial transmission [42]. Although Brazil began addressing the issue of DRTB in 1995 through research programs, national treatment protocols with free treatments, and epidemiological surveillance, social determinants of health only became a prominent topic in 2018, despite being recognized in the literature as a major concern [43].

Furthermore, while the universal health coverage provided by SUS has reduced health inequalities and aligns with strategies against TB, access and quality of healthcare services in Brazil remain a challenge, as they are widely recognized determinants of the burden of DRTB [44]. Consistent with the literature [1,10], the positive association we found between TB and HIV coinfection in DRTB, highlights some gaps in population health. Conversely, the protective characteristics of the Family Health Strategy coverage underscore the importance of health access in disease control.

However, the negative spatial autocorrelation between this indicator, primary care and healthcare agent coverage was not expected [10]. High healthcare coverage combined with an effective health surveillance, would likely improve DRTB case tracking. On the other hand, it could lead to better TB treatment outcomes, since the Brazilian guideline recommends DOT throughout treatment in partnership with primary care, which, in turn, would be responsible for monitoring treatment adherence, adverse effects, and complications, reducing the percentage of treatment loss to follow-up, an important cause of DRTB development [10,43,45]. It is important to mention that these results may reflect potential underreporting DRTB cases, a factor that may represent a gap in epidemiological surveillance and could bias our results.

Although 38 municipalities had no DRTB cases reported, it cannot be inferred that these areas are completely free of risk. The excess of zeros may reinforce the indication of underreporting, which is a concern endorsed by recent literature, highlighting a potential failure in health systems to capture all new cases [46,47]. Despite diagnostic and testing network of Brazilian guidelines efforts against TB, the unequal distribution of the Molecular Rapid Test for TB (MRT-TB or *GeneXpert*^®^), implemented in SUS since 2014, a test that diagnoses TB and can detect Rifampicin resistance - the main drug used in treatment - in less than an hour and a half, may increase the barriers of healthcare access [14,19].

The Rio de Janeiro state has the second large MRT-TB network in the country [14,19]. However, there is a centralization of tests machines in the metropolitan areas. According to spatial-temporal analysis, the city of Rio de Janeiro alone had a risk approximately 1.6 times higher from 2014 to 2019, and Duque de Caxias and São João de Meriti had similar risks from 2012 to 2016. This increased risk may be associated with the implementation and concentration of testing machines in these areas.

A study of the MRT-TB network in Rio de Janeiro state indicates that municipalities without machines request fewer test, potentially leading to underreporting cases in other regions [48]. It worth mentioning that the municipality of Belford Roxo, part of the high-risk DRTB cluster (high-high) in the metropolitan area found in our study, did not request MRT-TB test for any of its residents, indicating barriers of DRTB diagnosis access [48]. This demonstrates that the MRT-TB network in the state of Rio de Janeiro is still insufficient to capture DRTB cases, reinforcing concerns about primary DRTB infection. This situation is expected to improve with the new TB action plan aimed at deploying new machines [49].

The centralization of tests in the metropolitan area results in high population mobility to obtain MRT-TB, which is already established in the literature as one of the crucial factors in population health access and quality [5,7,50,51]. The BEL smoothing analysis reinforces the possible underreporting of cases, with a magnitude of the estimative greater than observed, considering the influence of neighbor’s municipalities on disease transmission. Similar results were found in national and international studies, indicating DRTB transmission and incidence related to spatial distribution [5,7,22].

Expanding access to faster diagnostics and drug susceptibility tests is essential to achieving national and global TB control goals [52]. The WHO End TB Strategy highlights the need for rapid diagnosis and universal testing, requiring a patient-centered diagnostic network with quality assurance. Improving patient access to diagnostic services and optimizing existing diagnostic capacity is essentially to an efficient and cost-effective system [53]. However, designing an excellent diagnostic network depends on multiple factors, including specific epidemiology, geography, demography, and local healthcare systems, which may vary within the country and different regions. These decisions have been made without systematic analysis to create evidence-based optimized diagnostic networks [54].

Additionally, the spatial autocorrelation between CIR and the proportion of the population aged 18 and over with completed Elementary school indicator was also an unexpected finding, indicating that DRTB increases as the proportion of the population with higher education increases. This diverges from the literature that stablished an association between lower education and TB incidence, as well as unfavorable TB and DRTB treatment outcomes [39,50]. Furthermore, education level is associated with income rates, which has a direct relationship with higher TB incidence and more frequent treatment loss to follow up [4,50,55].

It is important to highlight that the COVID-19 pandemic has reversed the worldwide progress in reducing the burden of TB and DRTB. Brazil was among the nations that observed a notable decline in reported cases from 2020 to 2021, potentially leading to an underreporting of cases in this study. This suggests that the number of untreated cases also increased during this period. In 2022, efforts to return cases reported to the normality began, however, the data reported in the SITE-TB included in this research continued to show a downward trend. Considering the increased social inequalities during the pandemic, enhancing access to healthcare, improving detection, and implementing strategies to promote adherence to DRTB treatment are imperative [1].

The results of this study should be analyzed according to its limitations. Challenges related to the incompleteness and quality of data from secondary databases are intrinsic to retrospective studies. It is relevant to highlight that SITE-TB, an information system focused on the management and clinical management of DRTB cases in Brazil, has non-standardized or specifically research-oriented information, which may impact the interpretation of results.

Another important limitation is the exclusive use of the SITE-TB database. Analysis with other databases that may record DRTB cases, such as SINAN, Laboratory Environment Manager (GAL), and Mortality System (SIM), was not done. This methodological choice may have omitted potential inconsistencies between databases, as demonstrated in previous studies, which demonstrated 25% underreporting cases between systems [47,56]. Furthermore, most demographic, socioeconomic, and healthcare access data were based on the 2010 CENSUS.

## Conclusion

This spatial analysis contributes to the knowledge of the spatial characterization of DRTB as well as for the identification of associated factors. The heterogeneous spatial distribution of DRTB cases, along with the positive spatial association between DRTB incidence and indicators, demonstrates that the TB and DRTB transmission dynamics can be explained by the perpetuation of social inequality and urban spatial organization. This highlights the importance of socio-spatial dynamics studies to control and prevention actions that are aligned with health necessities of populations and the syndemic profile. Additionally, smoothed rates BEG and BEL indicate that municipalities without reported cases are not excluded from risk, reinforcing the probability of underreporting DRTB cases. In addition to TB and DRTB control actions, such as promoting equity in healthcare access, which includes expanding and reorganizing the diagnostic and testing network, and providing comprehensive and integrated care in territories, it is crucial to implement concrete intersectoral policies focused on disease determinants. These policies should intervene in the social cycle of TB that perpetuates inequality and social exclusion, as determined by the National Tuberculosis Control Plan.

## Supporting information

S1 Table Description of indicators per data source and year. *Calculated by the author: Conducted using Software R version 4.3.0.
(DOCX)

S2 Table Aggregated information used in this study. (XLSX)

S3 Table Crude incidence rate and global and local empirical bayesian rate of municipalities of the Rio de Janeiro state, 2010–2022. (DOCX)
