# Supplementary material for: Spatial analysis of drug resistant tuberculosis (DRTB) incidence and relationships with determinants in Rio de Janeiro state, 2010 to 2022
Source: PLoS One. 2025 May 2;20(5):e0321553. doi: 10.1371/journal.pone.0321553 (PMC12047809; doi:10.1371/journal.pone.0321553)
Supplement: S1 Table — *Calculated by the author: Conducted using Software R version 4.3.0. (DOCX) [file pone.0321553.s001.docx]

**Supplementary material Table 1**. Description of indicators per data source and year

| **Dimension** | **Indicator** | **Definition** | **Source** | **Year** |
| --- | --- | --- | --- | --- |
| **Socioeconomics** | Municipal Human Development Index (MHDI) | Geometric mean of the Income, Education and Longevity dimension indices, with equal weights. | CENSUS | 2010 |
|  | GINI index | summary measure of income inequality | Atlas Brazil (PNUD, 2010) | 2010 |
|  | Percentage of population aged ≥18 years old with Elementary School completed | Ratio between the population aged 18 or over who completed Elementary School, in any of its modalities (Brazilian types of education: regular grade, non-grade, EJA or supplementary), and the total number of people in this age group, per 100. | CENSUS | 2010 |
|  | Percentage college degree completed population | Ratio between the population aged 25 or over who completed college degree and the total number of people in this age group, per 100 | CENSUS | 2010 |
|  | Percentage of poor population | Proportion of individuals with per capita household income equal to or less than R$ 140,00 per month in reais, in August 2010, per 100. | Atlas Brazil (PNUD, 2010) | 2010 |
|  | Proportion of people vulnerable to poverty | Proportion of individuals with per capita household income equal to or less than R$255.00 per month in reais, in August 2010, equivalent to 1/2 minimum wage on that date. | Atlas Brazil (PNUD, 2010) | 2010 |
| **Demographics** | Percentage of black/brown ethnicity | Calculated by the author*: Ratio between black/brown individuals and the total number of people per municipality, per 100. (This indicator has not presented current data by municipality level from CENSUS 2022 yet) | CENSUS | 2010 |
|  | Percentage of male gender | Calculated by the author*: Ratio between male individuals and the total number of people per municipality, per 100. (Data at municipal level only available until 2021) | Population estimative - IBGE | 2021 |
|  | Demographic density | Ratio between the total resident population and the area of ​​the municipality (inhabitant/km²). | CENSUS | 2022 |
|  | Household crowding density | The density of the household is given by the ratio between the total number of residents in the household and the total number of rooms used as bedrooms. | CENSUS | 2010 |
|  | Urban areas density | Based on a detailed study and interpretation of this methodology, IBGE classified dense urbanized areas, with dense areas being considered to be those with areas of continuous urban occupation that present greater proximity between buildings, with few empty or wooded spaces and large capillarity of roads. | Urban areas density - IBGE | 2019 |
| **Healthcare** | Percentage of TB treatment loss to follow-up | Calculated by the author*: Ratio of reported TB cases that were lost to follow-up during treatment by individuals with sensitive TB, multiplied by 100. | SINAN | 2010-2022  (Average) |
|  | Percentage of HIV-TB coinfection | Calculated by the author*: Proportion of notified TB cases that presented HIV co-infection by individuals with TB, per100. | SINAN | 2010-2022  (Average) |
|  | Family health strategy coverage | Average percentage of the population served by the family health strategy in relation to the population estimated (Data only available until 2020) | National Registry of Health Establishments (CNES) | 2010-2020 (Average) |
|  | Primary care coverage | Average percentage of the population registered in the Family Health and Primary Care Teams in relation to the population estimated. | National Registry of Health Establishments (CNES) | 2010-2022 (Average) |
|  | Healthcare agents’ coverage | Average percentage of households served by health agents in relation to the population estimated (Data only available until 2020) | National Registry of Health Establishments (CNES) | 2010-2020 (Average) |
|  | Percentage of drug susceptibility testing | Calculated by the author: Ratio between sensitivity tests for TB drugs and total TB cases according to municipality, per 100. | SINAN | 2010-2022 |

*Calculated by the author: Conducted using Software R version 4.3.0
