# Supplementary material for: Spatial analysis of drug resistant tuberculosis (DRTB) incidence and relationships with determinants in Rio de Janeiro state, 2010 to 2022
Source: PLoS One. 2025 May 2;20(5):e0321553. doi: 10.1371/journal.pone.0321553 (PMC12047809; doi:10.1371/journal.pone.0321553)
Supplement: S3 Table — (DOCX) [file pone.0321553.s003.docx]

**Supplementary material Table 3**. Crude Incidence Rate and Global and Local Empirical Bayesian Rate of municipalities of the Rio de Janeiro state,2010-2022.

| **Municipality** | **n** | **Population** | **Crude Incidence Rates (CIR)** | **Global Empirical Bayesian rates (BEG)** | **Local Empirical Bayesian rates (BEL)** |
| --- | --- | --- | --- | --- | --- |
| Angra dos Reis (RJ) | 5 | 167418 | 2.99 | 4.45 | 1.85 |
| Aperibé (RJ) | 2 | 11034 | 18.13 | 17.32 | 11.10 |
| Araruama (RJ) | 3 | 129669 | 2.31 | 4.23 | 3.34 |
| Areal (RJ) | 1 | 11828 | 8.45 | 13.71 | 2.31 |
| Armação dos Búzios (RJ) | 0 | 40006 | 0.00 | 5.57 | 5.38 |
| Arraial do Cabo (RJ) | 0 | 30986 | 0.00 | 6.56 | 5.20 |
| Barra do Piraí (RJ) | 3 | 92883 | 3.23 | 5.62 | 4.37 |
| Barra Mansa (RJ) | 14 | 169899 | 8.24 | 9.14 | 7.52 |
| Belford Roxo (RJ) | 48 | 483087 | 9.94 | 10.21 | 10.88 |
| Bom Jardim (RJ) | 0 | 28102 | 0.00 | 6.95 | 1.54 |
| Bom Jesus do Itabapoana (RJ) | 0 | 35173 | 0.00 | 6.06 | 6.98 |
| Cabo Frio (RJ) | 16 | 221987 | 7.21 | 8.00 | 6.34 |
| Cachoeiras de Macacu (RJ) | 1 | 56943 | 1.76 | 5.64 | 2.88 |
| Cambuci (RJ) | 0 | 14616 | 0.00 | 9.68 | 3.24 |
| Campos dos Goytacazes (RJ) | 37 | 483551 | 7.65 | 8.01 | 6.75 |
| Cantagalo (RJ) | 0 | 19390 | 0.00 | 8.50 | 1.52 |
| Carapebus (RJ) | 0 | 13847 | 0.00 | 9.91 | 2.96 |
| Cardoso Moreira (RJ) | 0 | 12958 | 0.00 | 10.18 | 6.92 |
| Carmo (RJ) | 2 | 17198 | 11.63 | 14.43 | 5.55 |
| Casimiro de Abreu (RJ) | 0 | 46110 | 0.00 | 5.05 | 2.96 |
| Comendador Levy Gasparian (RJ) | 0 | 8741 | 0.00 | 11.69 | 3.10 |
| Conceição de Macabu (RJ) | 0 | 21104 | 0.00 | 8.14 | 5.53 |
| Cordeiro (RJ) | 1 | 20783 | 4.81 | 10.68 | 1.05 |
| Duas Barras (RJ) | 0 | 10980 | 0.00 | 10.83 | 1.77 |
| Duque de Caxias (RJ) | 198 | 808152 | 24.50 | 24.32 | 24.41 |
| Engenheiro Paulo de Frontin (RJ) | 0 | 12242 | 0.00 | 10.40 | 5.84 |
| Guapimirim (RJ) | 3 | 51696 | 5.80 | 8.86 | 6.06 |
| Iguaba Grande (RJ) | 2 | 27920 | 7.16 | 11.18 | 4.29 |
| Itaboraí (RJ) | 31 | 224267 | 13.82 | 14.07 | 13.73 |
| Itaguaí (RJ) | 15 | 116841 | 12.84 | 13.42 | 20.42 |
| Italva (RJ) | 0 | 14073 | 0.00 | 9.84 | 6.77 |
| Itaocara (RJ) | 0 | 22919 | 0.00 | 7.80 | 0.77 |
| Itaperuna (RJ) | 7 | 101041 | 6.93 | 8.55 | 6.38 |
| Itatiaia (RJ) | 0 | 30908 | 0.00 | 6.57 | 7.17 |
| Japeri (RJ) | 12 | 96289 | 12.46 | 13.21 | 13.94 |
| Laje do Muriaé (RJ) | 0 | 7336 | 0.00 | 12.29 | 5.18 |
| Macaé (RJ) | 9 | 246391 | 3.65 | 4.63 | 2.78 |
| Macuco (RJ) | 0 | 5415 | 0.00 | 13.23 | 1.57 |
| Magé (RJ) | 21 | 228127 | 9.21 | 9.81 | 9.76 |
| Mangaratiba (RJ) | 0 | 41220 | 0.00 | 5.45 | 2.44 |
| Maricá (RJ) | 10 | 197300 | 5.07 | 6.14 | 7.45 |
| Mendes (RJ) | 1 | 17502 | 5.71 | 11.62 | 5.40 |
| Mesquita (RJ) | 20 | 167128 | 11.97 | 12.48 | 16.23 |
| Miguel Pereira (RJ) | 5 | 26578 | 18.81 | 17.99 | 17.65 |
| Miracema (RJ) | 0 | 26881 | 0.00 | 7.13 | 3.45 |
| Natividade (RJ) | 1 | 15074 | 6.63 | 12.43 | 5.03 |
| Nilópolis (RJ) | 19 | 146774 | 12.95 | 13.41 | 22.10 |
| Niterói (RJ) | 88 | 481758 | 18.27 | 18.21 | 19.51 |
| Nova Friburgo (RJ) | 3 | 189937 | 1.58 | 3.02 | 2.15 |
| Nova Iguaçu (RJ) | 126 | 785882 | 16.03 | 16.05 | 16.75 |
| Paracambi (RJ) | 4 | 41375 | 9.67 | 11.99 | 10.48 |
| Paraíba do Sul (RJ) | 1 | 42063 | 2.38 | 7.00 | 2.23 |
| Paraty (RJ) | 0 | 44872 | 0.00 | 5.15 | 2.36 |
| Paty do Alferes (RJ) | 1 | 29619 | 3.38 | 8.77 | 3.19 |
| Petrópolis (RJ) | 5 | 278881 | 1.79 | 2.79 | 2.38 |
| Pinheiral (RJ) | 0 | 24298 | 0.00 | 7.55 | 4.19 |
| Piraí (RJ) | 4 | 27474 | 14.56 | 15.53 | 8.36 |
| Porciúncula (RJ) | 1 | 17288 | 5.78 | 11.69 | 4.70 |
| Porto Real (RJ) | 0 | 20373 | 0.00 | 8.29 | 8.99 |
| Quatis (RJ) | 1 | 13682 | 7.31 | 12.95 | 7.69 |
| Queimados (RJ) | 18 | 140523 | 12.81 | 13.31 | 13.84 |
| Quissamã (RJ) | 0 | 22393 | 0.00 | 7.89 | 6.73 |
| Resende (RJ) | 15 | 129612 | 11.57 | 12.27 | 9.10 |
| Rio Bonito (RJ) | 2 | 56276 | 3.55 | 7.01 | 4.33 |
| Rio Claro (RJ) | 0 | 17401 | 0.00 | 8.96 | 5.30 |
| Rio das Flores (RJ) | 0 | 8954 | 0.00 | 11.60 | 1.31 |
| Rio das Ostras (RJ) | 7 | 156491 | 4.47 | 5.86 | 3.56 |
| Rio de Janeiro (RJ) | 1644 | 6211423 | 26.47 | 26.44 | 26.41 |
| Santa Maria Madalena (RJ) | 1 | 10232 | 9.77 | 14.45 | 6.82 |
| Santo Antônio de Pádua (RJ) | 0 | 41325 | 0.00 | 5.45 | 0.24 |
| São Fidélis (RJ) | 0 | 45059 | 0.00 | 7.37 | 6.45 |
| São Francisco de Itabapoana (RJ) | 1 | 38939 | 2.57 | 5.13 | 6.58 |
| São Gonçalo (RJ) | 155 | 896744 | 17.28 | 17.28 | 17.10 |
| São João da Barra (RJ) | 2 | 36573 | 5.47 | 9.46 | 6.85 |
| São João de Meriti (RJ) | 111 | 440962 | 25.17 | 24.82 | 25.07 |
| São José de Ubá (RJ) | 0 | 7070 | 0.00 | 12.42 | 3.51 |
| São José do Vale do Rio Preto (RJ) | 0 | 22080 | 0.00 | 7.95 | 2.21 |
| São Pedro da Aldeia (RJ) | 7 | 104029 | 6.73 | 8.34 | 5.61 |
| São Sebastião do Alto (RJ) | 0 | 7750 | 0.00 | 12.11 | 1.74 |
| Sapucaia (RJ) | 0 | 17729 | 0.00 | 8.88 | 3.15 |
| Saquarema (RJ) | 1 | 89559 | 1.12 | 3.96 | 3.18 |
| Seropédica (RJ) | 3 | 80596 | 3.72 | 6.30 | 12.31 |
| Silva Jardim (RJ) | 0 | 21352 | 0.00 | 8.09 | 1.80 |
| Sumidouro (RJ) | 0 | 15206 | 0.00 | 9.52 | 1.90 |
| Tanguá (RJ) | 0 | 31086 | 0.00 | 6.54 | 3.50 |
| Teresópolis (RJ) | 4 | 165123 | 2.42 | 3.96 | 2.05 |
| Trajano de Moraes (RJ) | 0 | 10302 | 0.00 | 11.08 | 2.59 |
| Três Rios (RJ) | 3 | 78346 | 3.83 | 6.45 | 2.18 |
| Valença (RJ) | 1 | 67753 | 1.48 | 4.94 | 3.93 |
| Varre-Sai (RJ) | 0 | 10207 | 0.00 | 11.11 | 2.57 |
| Vassouras (RJ) | 0 | 33976 | 0.00 | 6.19 | 1.68 |
| Volta Redonda (RJ) | 13 | 261584 | 4.97 | 5.80 | 5.36 |
